# ΔNp73 Enhances Promoter Activity of TGF-β Induced Genes

**DOI:** 10.1371/journal.pone.0050815

**Published:** 2012-12-07

**Authors:** Maarten Niemantsverdriet, Peter Nagle, Roland K. Chiu, Johannes A. Langendijk, Harm H. Kampinga, Robert P. Coppes

**Affiliations:** 1 Department of Cell Biology, Section Radiation and Stress Cell Biology, University Medical Center Groningen, University of Groningen, Groningen, The Netherlands; 2 Department of Radiation Oncology, University Medical Center Groningen, University of Groningen, Groningen, The Netherlands; Vanderbilt University Medical Center, United States of America

## Abstract

The p53 homolog p73 is frequently overexpressed in cancers. Especially the transactivation domain truncated isoform ΔNp73 has oncogenic properties and its upregulation is associated with poor patient survival. It has been shown that ΔNp73 has an inhibitory effect on the transactivation capacity of p53 and other p73 isoforms. Here, we confirm this finding but surprisingly find that ΔNp73 may also stimulate the expression of TGF-β signaling targets. Promoter-reporter analysis indicated that the presence of Smad Binding Elements (SBE) in the promoter is sufficient for stimulation of gene expression by ΔNp73. TGF-β signaling was less efficient in ΔNp73 downregulated cells, whereas tetracycline induced ΔNp73 increased expression of endogenous TGF-β regulated genes PAI-1 and Col1a1. Pull-down assays with SBE DNA suggest that ΔNp73 enhances smad3/4 binding to SBEs, thereby stimulating TGF-β signaling. Chromatin immunoprecipitation assays confirmed a direct interaction between ΔNp73 and SBE. Given the role of TGF-β signaling in carcinogenesis, tumor invasion and metastasis via targets like PAI-1 and Col1a1, our data suggest a model on how this effect of ΔNp73 could be a contributing factor in cancer progression.

## Introduction

The role of p53 homolog and family member p73 in carcinogenesis is not completely clear [1]–[3]. P53 is established as a transcription factor that functions as tumor suppressor. Mutations in the p53 gene occur in over 50% of tumors and play a major role in oncogenic transformation [2], [4], [5]. P73 has many transcriptional targets in common with p53 but its role in carcinogenesis is much more complex. This is partially due to the presence of multiple p73 variants as a result of multiple promoter usage and alternative splicing. Several variants (TAp73) are transcriptionally active but also inactive isoforms (ΔNp73) that lack the N-terminal transactivation domain may be expressed. TAp73 isoforms act as transcription factors and usually behave similar to p53 [1]. In contrast, ΔNp73 usually acts as inhibitor of transactivation competent p53 and TAp73, either by inactivating complexes containing active p53 family members or by competing for promoter binding sites [1], [6]. Mutations in the p73 gene are rarely found in tumors, in contrast to p53. However, it has been shown that changes in expression of specific p73 variants influences the carcinogenic character of tumors [2], [3]. P73 is upregulated in many cancer types and multiple studies show that high p73 expression correlates with poor patient survival [2]. The upregulation of especially the ΔNp73 variant has been linked with poor prognosis. Indeed, ΔNp73 is frequently overexpressed in tumors of the lung, breast, brain, thymus, colon, prostate, skin, ovary, muscle and other organs [3], [7]. The inhibitory effect of ΔNp73 on p53 and TAp73 variants has been suggested to be responsible for the oncogenic effect of ΔNp73 [2], [3]. The role for p73 carcinogenesis appears to arise solely from an imbalance between the isoforms since it was not observed in mice lacking p73 [6]. However p73 knockout mice did show growth defects, hippocampal dysgenesis and neurological, inflammatory and pheromonal deficiencies [6], thus emphasizing its role in development. Indeed unexpected biological effects (in for example neurogenesis) were discovered in p73-isoform specific mice [8]–[11] indicating that the interplay between the main p73 isoforms and the resulting biological impact may be much more complex than previously anticipated.

Multiple genes are regulated by a cooperation between p53 family members and TGF-β signaling, together inducing synergistic transcriptional activation [12], [13]. Interestingly, it has been hypothesized that like p53, p73 could also interact with TGF-β signaling [12], [14], [15] albeit through unknown mechanisms. As for p73, the TGF-β signaling pathway is essential for development. It is crucial for differentiation of embryonic tissue and morphogenesis of organs and is required for tissue homeostasis [16]. TGF-β signaling has both tumor suppressing and promoting activities [17]–[21]. During tissue homeostasis tumor suppressor activities of TGF-β dominate, whereas during tumorigenesis an increase in TGF-β signaling intensity may promote tumor progression [20], [21]. Although TGF-β signaling controls many different actions in many different cell types, the diversity of its response is generated by reacting in divergent ways to essentially the same signaling cascade. After binding of TGF-β or its homologs to the TGF-β receptors, Smad2 and/or Smad3 are activated, bind Smad4 and translocate to the nucleus where they bind and activate promoters with Smad Binding Elements (SBE). Because the DNA binding capacity of Smads alone is very low, additional site-specific transcription factors are required for a full TGF-β response [18], [22]. As such p53 can bind a promoter's p53 Binding Element and together with TGF-β cooperatively enhance expression of specific genes [12]. Considering the similar roles of p73 and TGF-β signaling, a connection between these two could have strong implications for the understanding of both development and carcinogenesis. Next to these, also the PAI-1 gene has roles in many biological processes including development and carcinogenesis [23]–[25] as it is a key negative regulator of the plasmin system of extracellular matrix proteases. Interestingly, PAI-1 expression is regulated by both TGF-β and p53 [12], [26], [27]. Therefore we started to investigate a possible connection between p73 and TGF-β signaling by using PAI-1 reporter constructs available in our lab previously used to study TGF-β signaling, p53 and their cooperation [26], [27]. In the present study, we report that several p73 isoforms, like p53, indeed can modulate TGF-β signaling. Unexpectedly, however, the ΔNp73 variant that normally antagonizes p53/p73 effects had the largest boosting effect on TGF-β mediated target activation. Our results indicate that ΔNp73 may directly increase the transactivation of TGF-β signaling targets in a non-canonical manner, potentially by forming a complex with Smads on Smad Binding Elements.

## Materials and Methods

### Plasmids

The PAI-luc, PAI p53-M luc and SBE-luc reporter constructs and pcDNAp53 were described previously in Hageman *et al.* [26]. The P21^Waf^-Luc reporter construct was described in [28] pcDNASmad3 and pcDNASmad4 were kindly provided by Dr. B. Eggen (Haren, The Netherlands). pcDNASmad7 plasmid [29] was a kind gift of Dr. C-H. Heldin (Uppsala, Sweden). Plasmids containing p73 splice variants [30], [31] were kindly provided by Dr. Gerry Melino (Rome, Italy). pcDNA3.1+ was purchased from Invitrogen. Smad2 expression was done with pGFPSmad2, which was constructed by ligating a XhoI-EcoRI digested PCR product of primers 5′- CGCACTCGAGGGATGTCGTCCATCTTGCCATTCAC and 5′-CGCGAATTCTTATGACATGCTTGAGCAACGCAC into the EcoRI-XhoI digested pEGFP-C1 plasmid (Clontech). pYFPSmad4 was constructed by ligating an EcoRI-XhoI digested PCR product of primers: 5′-GCGCTCGAGGGATGGACAATATGTCTATTACGAATACACC and 5′-GCGGAATTCTTCAGTCTAAAGGTTGTGGGTCTG into EcoRI-XhoI digested pEYFP-C1 plasmid (clontech). The pV5Smad3 plasmid was created by first creating pCFPSmad3 by ligating a XhoI-EcoRI digested PCR product of 5′-GCGCTCGAGGGATGTCGTCCAT CCTGCCTTTCA and 5′-CGCGAATTCTAAGACACACTGGAACAGCG into the XhoI-EcoRI sites of pECFP-C1 (Clontech). To create pV5Smad3, pCFPSmad3 was digested with NheI-XhoI (Cuts out ECFP) and the gap was filled up with a linker of a duplex of 5′- CTAGTATGGGTAAGCCTATCCCTAACCCTCTCCTCGGTCTCGATTCTACGCATCATCACCATCACCATGC (upper oligo) and TCGAGCATGGTGATGGTGATGATGCGTAGAATCGAGACCGAGGAGAGGGTTAGGGATAGGCTTACCCATA.

 pcDNA5 FRT TO ΔNp73α was constructed by ligating the HindIII-XhoI ΔNp73α fragment of pcDNAHAΔNp73α into pcDNA5 FRT TO (Invitrogen) digested with HindIII-XhoI. All newly constructed plasmids were verified by sequencing.

### Cell culture and construction of tetracycline regulated ΔNp73 cell line

Hep3B cells (ATCC) were described to be p53 and p73 negative [32] and were cultured as described [26]. Smad4 deficient MDA-MB-468 cells were cultured as described in [33]. Transfections were done with lipofectamine (Invitrogen) in 24 wells plates that were 60% confluent at the time of transfection, the method was basically as described [26]. Hek293 cells used were Flp-In T-Rex HEK293 cells purchased from Invitrogen. The Hek293ΔNp73 cell line was constructed as suggested by the manufacturer, using the pcDNA5 FRT TO ΔNp73α plasmid. Unless mentioned otherwise Hep3B cells were used.

### Immunoblotting

Sample preparation and blotting was following standard procedures as described [28]. Membranes were reacted with the following primary antibodies: α-V5 (Invitrogen) for V5-tagged Smad3, α-HA (Covance) for HA-tagged p73, PAN-p73 IMG-259A (Imgenex), α-GFP (Santa Cruz) for tagged Smad4, and α-Smad3 clone 2C12 (Sigma-Aldrich) for endogenous Smad3, followed by the appropriate HRP-conjugated secondary antibody and ECL detection.

### Luciferase assays

**Figure 1:**
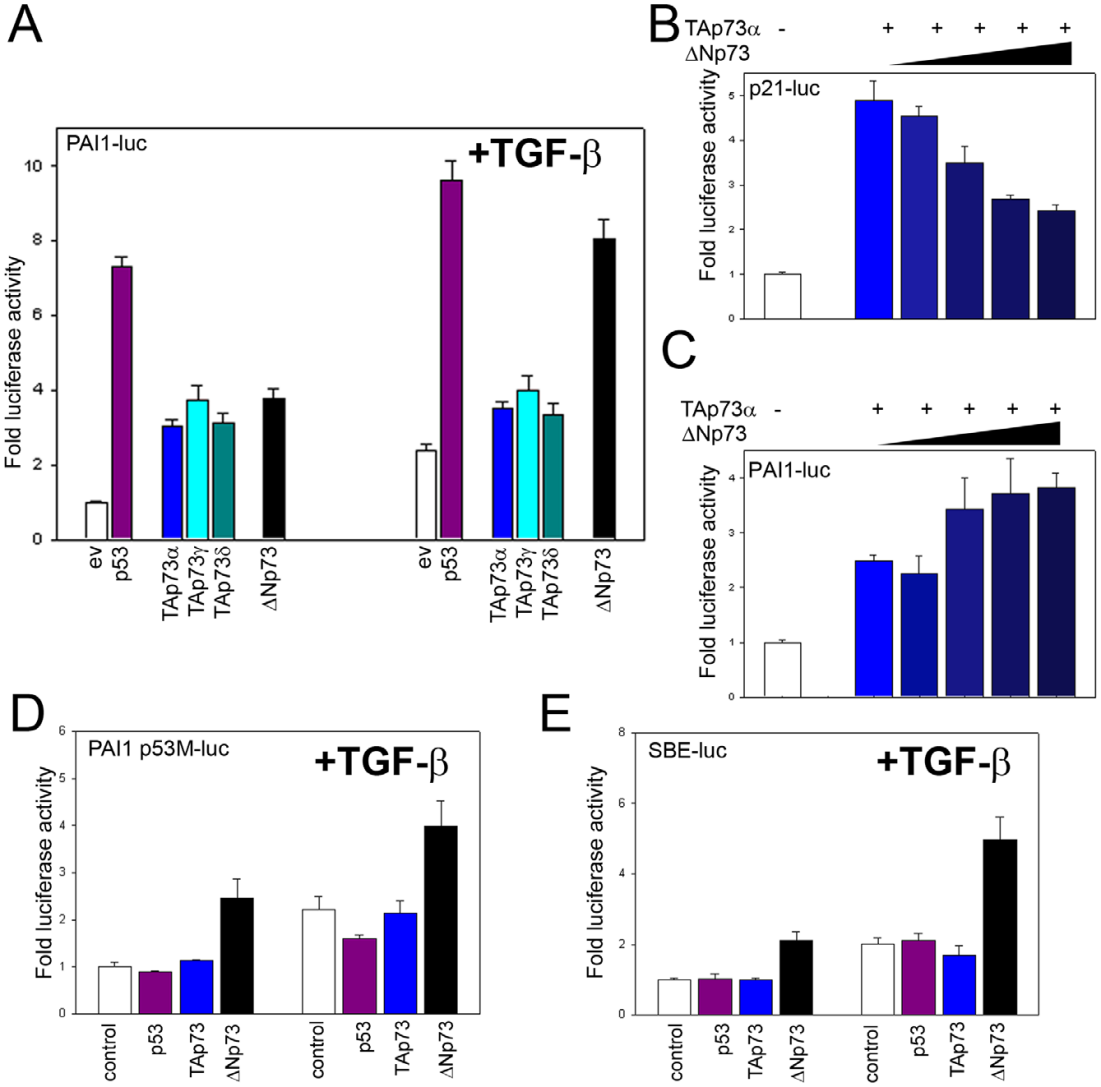
ΔNp73 enhances promoter activity via Smad Binding Elements in Hep3B cells that lack expression of endogenous p73 and p53 a) Induction of PAI-1-luc by p53 or p73 variants and/or TGF-β1 after transfection. control = empty vector. b) Induction of p21-luc by TAp73α and its inhibition by co-expression of increasing amounts of ΔNp73. c) Induction of PAI-1-luc by TAp73α and its enhancement by co-expression of increasing amounts of ΔNp73. d) Activation of the PAI-1-luc promoter with a mutated p53 Binding Element: induction of promoter activity by p53 and TAp73 depends on an intact p53 binding element in the promoter, whilst ΔNp73 shows activity even if the p53 binding element is lacking. Transfected cells were cultured in the presence of 1 ng/ml TGF-β1 for 24 hours where indicated (right). e) Induction of Smad Binding Elements by p53 or p73 variants and/or TGF-β. Only ΔNp73 shows activity. Transfected cells were cultured in the presence of 1 ng/ml TGF-β1 for 24 hours. control = empty vector. TAp73γ and δ forms are shown only in Figure 1a and were omitted in the rest of the figures for simplicity; they always showed similar results as TAp73.

Luciferase assays were performed as described [26]. Cells grown on 24-wells plates were co-transfected using lipofectamine (Invitrogen) with 0.5 µg reporter plasmid per well and 10 ng (or other where indicated) empty vector or p53/73 expression plasmid. For Figures 1b and c, 0,5 µg reporter plasmid (PAI-luc or p21-luc) was co-transfected with 4 ng pHATAp73α and 0, 2, 4, 8 and 16 ng pHAΔNp73α, total plasmid amounts and volumes were equalized in every experiment by adding pcDNA3.1+. In all assays where only TAp73 or ΔNp73 is mentioned, the α splice-variants were used. Unless stated otherwise, luciferase assays were performed in Hep3B cells.

### DNA Affinity Immunoprecipitation (DNAP)

For DNAP experiments with transfected cells, 50% confluent Hek293 cells on 6 cm dishes were transfected with the indicated plasmids using lipofectamine (invitrogen). 24 hours post transfection TGF-β1 (1 ng/ml) was added to some of the dishes. 24 hours later, cells were trypsinized, washed once with cold PBS and the pellet was taken up in 600 µl cold DNAP buffer (10 mM Tris-HCl, pH 7.4, 150 mM NaCl, 1 mM EDTA, 1% Nonidet P-40) with 1 mM sodium Vanadate, 0.4 mM PMSF, 50 mM NaF and complete protease inhibitors (Boehringer). Cells were passed through a 26 GA needle several times and lysates were centrifuged at 4C for 15 minutes in an Eppendorf centrifuge at maximum speed. 550 µl supernatant was transferred to a new tube. 50 µl was transferred to another new tube, 50 µl 2× protein sample buffer was added and these samples were boiled for 5 minutes to obtain input fractions. To the remaining 500 µl lysate, 5 µg Poly(dI-dC) (Sigma-Aldrich) was added and lysates were tumbled slowly (10 RPM) for 30 minutes. 500 pmol of biotinylated oligo duplexes with the sequences 5′-AGACAGACAGACAGACAGACAGACAGACAGAC and 5′-GTCTGTCTGTCTGTCTGTCTGTCTGTCTGTCT (Smad Binding Elements) were added and lysates were tumbled again for 30 minutes. 50 µl of 6% conjugated Streptavidin-agarose bead slurry (Pierce) was added and lysates were tumbled for another 4 hours. Beads were collected by centrifugation for 30 seconds at 6500RPM in an Eppendorf centrifuge and washed 4 times with 1 ml DNAP buffer each wash, after this the oligos were either incubated with another cell lysate or buffer or processed immediately. After the last wash 50 µl fluid was left on the beads and 50 µl 2× sample buffer was added, samples were boiled for 5 minutes. 10 µl of input or beads was separated on a PAGE gel and transferred to nitrocellulose membrane.

### QPCR

Quantitative PCR for PAI-1 was performed as described in [26] using the same primersets. Col1a1 was detected using the same method with primers: fwd 5′-CAATGCTGCCCTTTCTGCTCCTTT and reverse: 5′- CACTTGGGTGTTTGAGCATTGCCT.

### Chromatin immunoprecipitation (ChIP)

Hep3B cells were transiently transfected with pcDNA5 FRT TO ΔNp73α plasmid at a concentration of 1 µg/ml in 10 cm dishes using Lipofectamine (Invitrogen) according to the manufacturer's protocol. The media was supplemented with TGF-β1 (1 ng/ml) 24 h after transfection. A further 24 h later, the cells were fixed by replacing the culture media with 1% formaldehyde in PBS and incubated for 10 minutes at room temperature. Fixation was stopped by the addition of glycine (0.125 M final) for 5 minutes. Fixed cells were washed once with PBS and harvested in SDS Buffer (50 mM Tris pH 8.1, 0.5% SDS, 100 mM NaCl, 5 mM EDTA, 0.02 % NaN_3_ and protease inhibitors). Cells were centrifuged at 1000 RPM for 5 minutes and the cell pellets were resuspended in 3 mL IP buffer (100 mM Tris pH 8.6, 0.3 % SDS, 1.7 % Triton X-100, 0.02 % NaN_3_ and 5 mM EDTA). Sonication was performed using a Branson Sonifier B-12, yielding genomic DNA of a bulk size of 200–1000 bp. For each immunoprecipitation, 0.5 mL of lysate was precleared by the addition of 15 µl of Protein A/G beads (Santa Cruz). Immunoprecipitation was performed by incubating overnight at 4°C with antibodies for HA-tag (Covance) and mouse control IgG ChIP Grade (Abcam; ab18413) at final concentrations of 4 µg/mL on a rotator. Antibody bound samples were recovered with 20 µl protein A/G beads incubated at 4°C on rotating wheel for 4 hours. Beads were washed with successive washes of Mixed Micelle Buffer (150 mM NaCl, 20 mM Tris-Cl pH 8.1, 5 mM EDTA pH 8.0, 5.2% w/v sucrose, 0.02% NaN_3_, 1% Triton X-100, 0.2% SDS), Buffer 500 (0.1% w/v deoxycholic acid, 1 mM EDTA pH 8.0, 50 mM Hepes pH 7.5, 1% Triton X-100, 0.02% NaN_3_), LiCl Detergent Wash Buffer (0.5% w/v deoxycholic acid, 1 mM EDTA pH 8.0, 250 mM LiCl, 0.5% NP-40, 10 mM Tris-Cl pH 8.0, 0.02% NaN_3_) and TE Buffer. Complexes were eluted in Elution Buffer (1% SDS, 0.1 M sodium bicarbonate) at 65°C in a shaking incubator for 2 hours followed by reversal of the cross-links by overnight incubation at 65°C. DNA was isolated using Nucleic Acid and Protein Purification kit (Macherey-Nagel) following the manufacturer's instructions and resuspended in 60 µl elution buffer and further diluted to 280 µl with TE Buffer.

Quantitative PCR was performed using the following primer sets for PAI-1: fwd 5′-CCTCCAACCTCAGCCAGACAAG and rev 5′-CCCAGCCCAACAGCCACA, p21: fwd 5′-ACTTGTCCCTAGGAAAATCC and rev 5′-GAAAACGGAGAGTGAGTTTG, and Col1a1 fwd 5′-CAGAGCTGCGAAGAGGGGA and rev 5′-AGACTCTTTGTGGCTGGGGAG. Primers specific for the promoter region of PTEN were used as a positive control for the assay and their sequences were fwd 5′-ATGTGGCGGGACTCTTTATG and rev 5′-CGCGCTCAACTCTCAAACTT.

## Results

### ΔNp73 oppositely affects PAI-1 and p21^WAF^ promoter activation

To examine a potential cooperation between different p73 variants and TGF-β signaling, we used Hep3B cells that do not express endogenous p53 or p73 (Figure S1 and [26], [32]) and express modest levels of ectopic DNA (Figure S2A). First, we transfected a PAI1 promoter-luciferase construct (PAI1-luc) and studied the induction of the PAI-1 gene, a gene that plays an important role in development and carcinogenesis [23]–[25], [34] and which is known to respond to both p53 and TGF-β [12], [26], [27]. This PAI-1 promoter contains a p53 binding-element and several Smad Binding Elements [12], [26], [35]. As expected [12], [26], [27], co-transfection of PAI1-luc with a low concentration of a plasmid expressing p53 strongly increased luciferase activity (Figure 1A, left). Similarly, also the transactivation competent p73 isoforms TAp73α, TAp73γ and TAp73δ induced PAI-1 reporter gene expression (Figure 1A, left). Surprisingly, the ΔNp73 form normally acting as inhibitor of p53 and TAp73 [1], [6], also increased luciferase activity indicative of activation of the PAI-1 promoter. In the presence of TGF-β1, known to cooperate with p53 in PAI-1 transactivation [26], [27], luciferase activity was even further enhanced (Figure 1A, right). In contrast, luciferase activity induced by the other TAp73 variants was not further enhanced by TGF-β1.

To check whether our ΔNp73 construct was capable to perform its described inhibitory activity, we investigated the ability of ΔNp73 to antagonize TAp73α induced activation of the p21^WAF^ promoter, a well characterized target of the p53 family [1], [31], [36]. In agreement with reports by others [31], [37], [38], ΔNp73 dose-dependently reduced TAp73α induced activation of a p21^WAF^ promoter reporter gene (Figure 1B). However, the reverse effect was seen for the PAI-1 promotor reporter gene, where ΔNp73 clearly increased promoter activation (Figure 1C) in line with the results presented in Figure 1A. In these experiments, cells were transfected with very small amounts of p53 family members and expression levels of ectopic p73 in Hep3B cells appeared not to be exceptionally high under the experimental conditions used (Figure S2A). Since these assays rely on ectopic expression of ΔNp73, one could argue that they may therefore not reflect physiological conditions. However, high local levels of ΔNp73 have been observed in specific parts of the mouse brain during early development [6] and ΔNp73 expression levels of up to 150 fold of that of normal tissue have been observed in aggressive tumors indicating biological relevance of our data (reviewed in [38]).

### ΔNp73 activity does not depend on the p53 Binding Element but on Smad Binding elements

Next we investigated whether the effect of ΔNp73 on PAI-1 expression was dependent on the binding to the p53 binding element in the PAI-1 promotor. Indeed. as expected transfection of p53 and TAp73 did not increase activation of a PAI-1 promoter luciferase construct with a mutated p53 binding element (Figure 1D) [26]. Interestingly, ΔNp73 still could activate the promoter lacking p53BE (Figure 1D). This implies that induction of PAI-1 expression by ΔNp73 must be due to effects on regulatory elements other than the p53BE. The PAI-1 promoter also contains binding elements for the transcription factors AP-1, SP-1, CRE and three Smad binding elements [26]. Since our previous work indicates that AP-1, SP-1 and CRE sites are not involved in the p53 or TGF-β induced stimulation of PAI-1 [26], [27], we hypothesized that the mechanism by which ΔNp73 induces expression of PAI-1 could depend on Smad signaling. To test this we used a promoter comprised exclusively of repeats of Smad Binding Elements (SBE-luc), which is routinely used to directly study TGF-β responses because there is no interference of non-TGF-β signaling regulated transcription factors [26], [39]. ΔNp73 could clearly induce this construct (Figure 1E), indicating that ΔNp73 might indeed affect TGF-β signaling whereas p53 or TAp73 did not. Hep3B cells under normal conditions do not express ΔNp73 (Figure S1) but do still have functional TGF-β signaling. Therefore, our observations indicate that ΔNp73 might act as an enhancer of basal TGF-β signaling, but is not absolutely required for TGF-β signaling.

### ΔNp73 stimulation of TGF-β signaling is cell type independent

**Figure 2:**
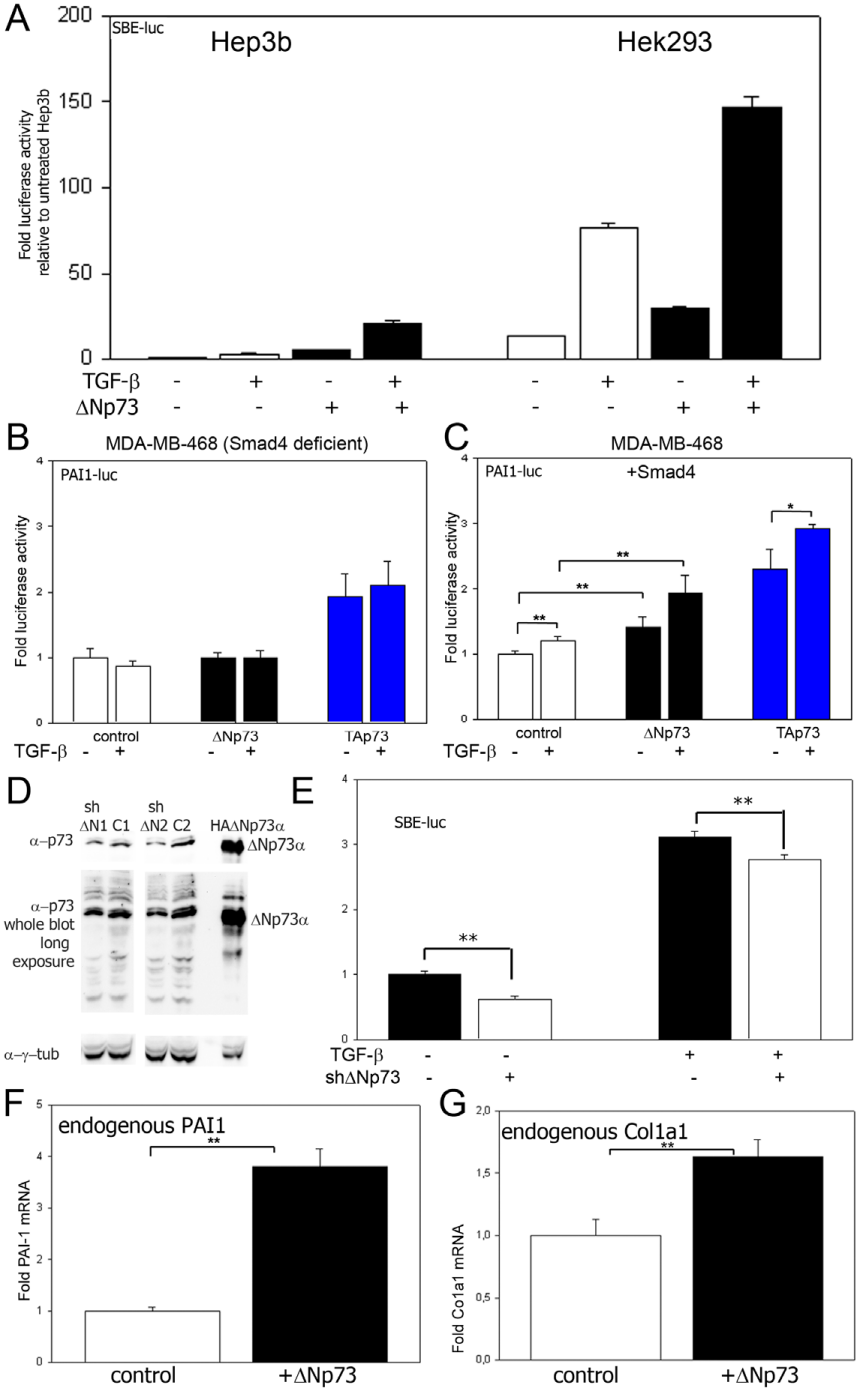
Stimulation of TGF-β signaling by ΔNp73 in Hep3B, Hek293 and MDA-MB-468 cells. a) Luciferase assays comparing the effect of ΔNp73 on Hep3B cells versus Hek293 cells using SBE-luc as reporter, cotransfected with either empty vector or 10 ng/well ΔNp73, and/or treated with 1 ng/ml TGF-β1. b) Luciferase assay of MDA-MB-468 cells (deficient for Smad4) transfected with 400 ng/well PAI1-luc, 100 ng/well empty vector (to compensate for Smad4 plasmid in Figure 2c) and either 10 ng/well empty vector (control), TAp73 or ΔNp73. Cells were grown with or without TGF-β1. c) Luciferase assay of MDA-MB-468 cells (deficient for Smad4) transfected with 400 ng/well SBE-luc, 100 ng/well Smad4 and 10 ng/well of either empty vector (control) or ΔNp73. Cells were grown in the presence of TGF-β1 where indicated. ** p<0.05 and *p<0.10 in a two-tailed T-test. d) Immunoblot analysis using a PAN-p73 specific antibody using lysates of Hek293 cells transfected with either empty pSuper vector as control (C1 or C2) or pSuper vectors expressing shRNA directed to the ΔN specific part of ΔNp73, a lysate of Hek293 cells transfected with a plasmid expressing HA-tagged ΔNp73α was used to serve as marker for the height of ΔNp73α. The bands corresponding to this height are shown additionally with a light balance appropriate for this band. Two ΔN p73 targeting sequences were used: ΔN1 and ΔN2. The PAN-p73 antibody detected multiple bands including the ΔNp73α variant. However, only a few specific bands, including a band with the height of ΔNp73α, were reduced whereas other bands were not affected, indicating that ΔNp73 variants were specifically downregulated. e) Luciferase assays of Hek293 cells transfected with SBE-luc and either pSuper empty vector control or pSuper ΔN1 and ΔN2 (combination), showing that (partial) ΔNp73 specific downregulation significantly decreases TGF-β signaling. ** p<0.05 in a two-tailed T-test. f) QPCR analysis of PAI-1 mRNA in tetracycline regulated ΔNp73 expressing cells, left untreated or after induction of ΔNp73. g) QPCR analysis of Col1a1 mRNA in tetracycline regulated ΔNp73 expressing cells left untreated or after induction of ΔNp73. ** p<0.05 in a two-tailed T-test.

It could be that the observed effects of ΔNp73 on TGF-β signaling are limited to Hep3B cells without p53 and p73 expressing moderate levels of transfected DNA. Therefore we tested Hek293 cells which express high levels of endogenous p53 and p73 variants (Figure S1) and efficiently express high levels of proteins from transfected DNA (Figure S2B and S3). Indeed also Hek293 cells showed a clear increase in SBE-luc activation after transfection with ΔNp73 as well as an additional increase in combination with TGF-β1 (Figure 2A), similar to Hep3B cells. These results indicate that ΔNp73 induced increase of TGF-β signaling is not cell-type restricted.

Although the common view is that the dominant-negative function on p53 dependent activities is the main function of ΔN p53 family members [40], positive effects of ΔN p53 family members on target gene expression have also been described [41]–[43]. Especially for ΔNp63 it is clear that it can act as positive regulator of target genes [41], [42]. However, for ΔNp73 it remains an enigma how it could activate the SBE-driven promoter. We hypothesized that it may enhance the activity of Smad proteins, the classical TGF-β-induced transcription factors [18], [22]. A central element in the TGF-β signaling routes is the Smad4 protein [18]. To test whether the increase of TGF-β signaling targets by ΔNp73 indeed depends on Smad signaling, we used a cell line that lacks the expression of Smad4 (MDA-MB-468 cells) and therefore is completely deficient for core TGF-β signaling [44], [45]. Because plasmid uptake was extremely low in these cells during transfection (Figure S2), the SBE-luc reporter could not be used. We therefore used the PAI1-luc reporter to detect a TGF-β signaling response. This promoter has a much higher read-out because it contains a comprehensive promoter with many transcription factor binding-elements [26], [46] in contrast to the SBE-luc promoter which consists simply of a stretch of SBE elements [26], [39]. Neither ΔNp73 nor TGF-β1 alone were capable of increasing PAI-1 induction in the MDA-MB-468 cells (Figure 2B), as would be expected when the activity of ΔNp73 would be SMAD dependent. Yet, TAp73 still could activate the PAI-1 promoter (Figure 2B), indicating that even with low transfection efficiencies our model works in this cell line. When Smad4 was reintroduced in MDA-MB-468 cells by transfection, PAI-1 induction was highly significantly increased by ΔNp73 only. The effect was further enhanced after addition of TGF-β1 (Figure 2C). It must be stated that the magnitude of effects seen were relatively small, but this is likely due to an only partial TGF-β signaling restoration related to the low transfection efficiency in these cells, which yield only low expression levels of Smad4 to a level far below the endogenous Smad4 levels of Hek293 cells (Figure S4). Nevertheless, even in these partially TGF-β signaling restored cells, the response to ΔNp73 was already highly significant (Figure 2C), suggesting biological significance.

To get an indication whether the ΔNp73 induced increase in TGF-β signaling might also be relevant under normal cellular conditions, endogenous ΔNp73 was down regulated in Hek293 cells that express ΔNp73 endogenously (Figure 2D), by shRNA designed to the ΔN specific sequence of ΔNp73. Because the ΔN specific part of ΔNp73, required for specific downregulation is very small, choices for shRNA were limited. Yet two target sequences partially down regulated ΔNp73 (Figure 2D). However, even with these limitations, the partial down regulation of ΔNp73 resulted in a significant decrease in SBE-luc promoter activity (Figure 2E). This indicates that at least some biological effect of ΔNp73 can be expected in influencing TGF-β signaling under normal cell conditions in Hek293 cells.

To mimic the high expression levels in aggressive tumors [38] and developing mouse brain [6], we investigated whether tetracycline-induced ΔNp73 (Figure S5) was capable of inducing the expression of endogenous target genes in Hek293 cells. Indeed, tetracycline-induced ΔNp73 expression increased endogenous PAI-1 mRNA in these cells (Figure 2F). Also the mRNA levels of another endogenous TGF-β signaling target gene, Col1a1 is a collagen component which has been shown to play a significant role in carcinogenesis [47]–[49] and that has a promoter containing validated SBEs [50] was significantly increased by ΔNp73 (Figure 2G). The induction of Col1a1 by ΔNp73 was verified by qPCR (data not shown). Together these data suggest that ΔNp73 expression can induce more than one endogenous TGF-β signaling target and that suggest that this mechanism works in different cell types.

### ΔNp73 and Smads cooperate in TGF-β signaling

**Figure 3:**
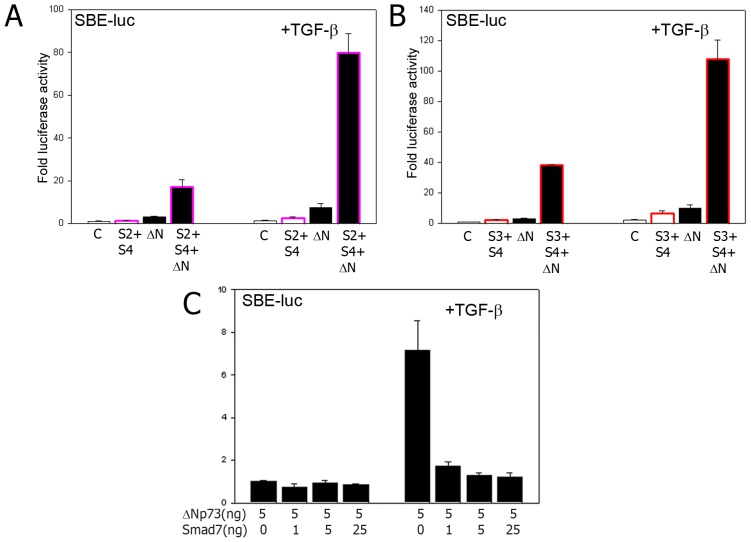
ΔNp73 and Smads cooperate in TGF-β signaling. A) Activation of the SBE-luc promoter by a Smad2+Smad4 combination (pink edged bars) and/or ΔNp73 (Black bars) and/or TGF-β1 (right panel). c = empty vector control. B) Activation of the SBE-luc promoter by a Smad3+Smad4 combination (red edged bars) and/or ΔNp73 (black bars) and/or TGF-β1 (right panel). C) Luciferase assay of cells transfected with SBE-luc reporter and ΔNp73 in combination with increasing amounts of (inhibitory) Smad7. Cells in the right panel were also treated with 1 ng/ml TGF-β1.

To further study the possible cooperation between Smad proteins and ΔNp73 in enhancing TGF-β signaling, ΔNp73 was expressed ectopically in combination with Smad2 or 3 and Smad4 in (TGF-β1 treated) Hep3B cells. When using a combination of either Smad2 or Smad3 together with Smad4, the activation of the SBE-luc construct was significantly enhanced, but when ΔNp73 was added, the SBE activity increased to extremely high levels (Figure 3A and B, note the fold increase of around 100 fold after TGF-β1 treatment) as occurs in human tumors [38]. In line with the hypothesis that Smad2/3 signaling is responsible for the induction of SBE containing promoters by ΔNp73, Smad7 which inhibits Smad2 and Smad3 activation [17], already at a very low concentration effectively prevented the ΔNp73 stimulated increase in TGF-β signaling in Hep3B cells (Figure 3C). This further suggests that ΔNp73 enhanced the core TGF-β pathway and required signaling by Smad proteins in our model.

### ΔNp73 and Smads form a complex with SBEs in vitro

**Figure 4:**
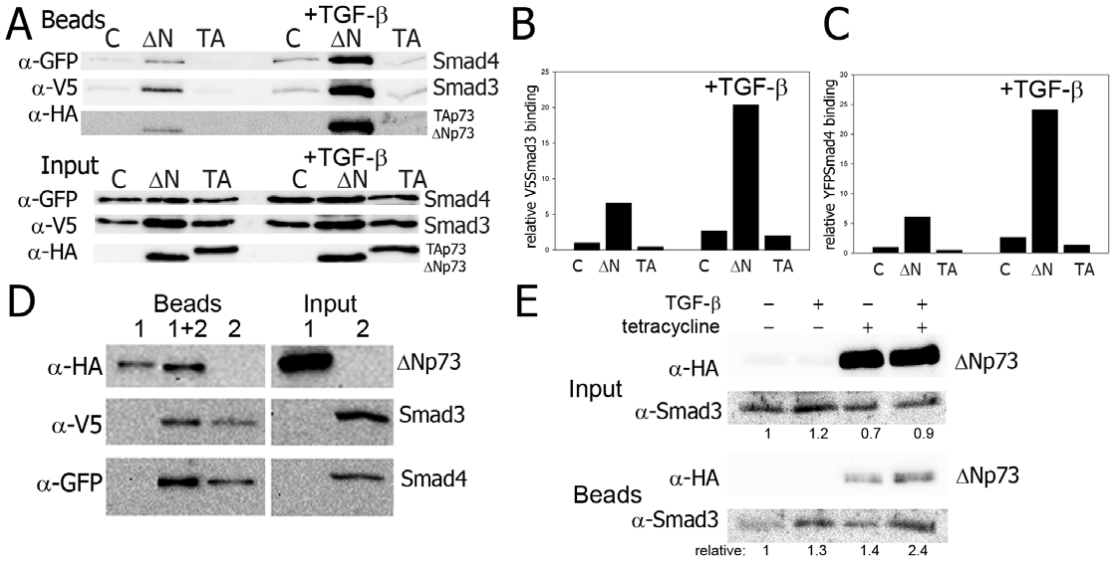
ΔNp73 and Smads form a complex with SBEs in vitro. A) DNA Affinity Precipitation assay (DNAP) of SBE DNA duplexes with extracts of cells left untreated or treated with TGF-β1 after triple transfection with YFP-tagged Smad4, V5-tagged Smad3 and either empty vector control (c), HA-tagged ΔNp73 (ΔN) or HA-tagged TAp73α (TA). B) Relative binding of V5Smad3 to SBE oligos, quantification and normalization for input values of the V5Smad3 results from Figure 3D. C) Relative binding of YFPSmad4 binding to SBE oligos, quantification and normalization for input values of the YFPSmad4 results from Figure 3D. D) Pull-down of SBE oligo incubated with a cell lysate containing HA-ΔNp73 (extract 1) or with a cell-lysate containing V5Smad3 and YFPSmad4 (extract 2) or incubated first with extract 1, washed and incubated with extract 2 (1+2). E) Immunoprecipitation (IP) with α-HA of HA-ΔNp73 induced cells (tetracycline) detecting endogenous Smad3.

To study if direct binding of Smad4 and/or Smad3 to the Smad binding elements might be involved, we determined the effect of ΔNp73 on the efficiency of binding of (tagged) Smad3 and Smad4 to oligos consisting of Smad Binding Elements. Using the DNA Affinity Precipitation (DNAP) assay, biotin-labeled oligos consisting of SBE repeats were incubated with protein extracts of untreated or TGF-β1 treated Hek293 cells. These cells ectopically expressed tagged Smad 3 and 4 proteins in combination with ΔNp73, TAp73 or without p73. We used Hek293 cells because they express high levels of transfected protein (Figure S2B), which is required for efficient DNAP. Extracts of unstimulated cells showed increased binding of Smad3 and Smad4 to DNA in the presence of ΔNp73. Adding TGF-β1 further increased Smad binding (Figure 4A). In contrast the presence of TAp73 did not increase Smad binding nor did it change the relative binding compared to control and TAp73 transfected cells in TGF-β treated cells (Figure 4 A, B and C). Intriguingly, ΔNp73 itself was also detected in large amounts in the DNA precipitate (Figure 4A). Therefore it potentially could be part of the Smad-SBE DNA complex. In line with the effects with ectopically co-expressed Smads, ΔNp73 also enhanced SBE binding of endogenous Smad3 (Figure 4E). However, interactions between ΔNp73 and endogenous Smad3 proteins in extracts of soluble proteins were not detected in the absence of DNA (Figure S6). In a subsequent DNAP experiment, protein extracts from ΔNp73 expressing cells or buffer were incubated with SBE oligos. The same oligos and bound proteins were next incubated with either buffer or a protein extract of TGF-β1 treated cells ectopically expressing both V5-tagged Smad3 and GFP tagged Smad4 (lysate 2) and pulled-down. As expected, Smad3 and Smad4 bound the SBE oligo (Figure 4D, beads 2). ΔNp73 itself was also pulled-down with the SBE DNA duplexes (Figure 4D, beads 2). It is unclear if this initial ΔNp73 binding occurs directly or through binding of endogenous Smads. However, pre-incubation of SBE oligos with extracts of ΔNp73 expressing cells bound more ectopic Smad3 and Smad4 than control SBE oligos (Figure 4D, beads compare 1+2 to 2). Inversely, ΔNp73 pull-down was more efficient with Smad3 and Smad4 incubated SBE oligos (Figure 4D, beads, compare 1+2 and 1) suggesting that Smad3/Smad4 and ΔNp73 mutually facilitate or stabilize each other's binding to the SBE DNA. ΔNp73 might bind to endogenous promoter DNA as seen for the PTEN promoter [51].

### ΔNp73 directly interacts with DNA at the Smad Binding Elements

**Figure 5:**
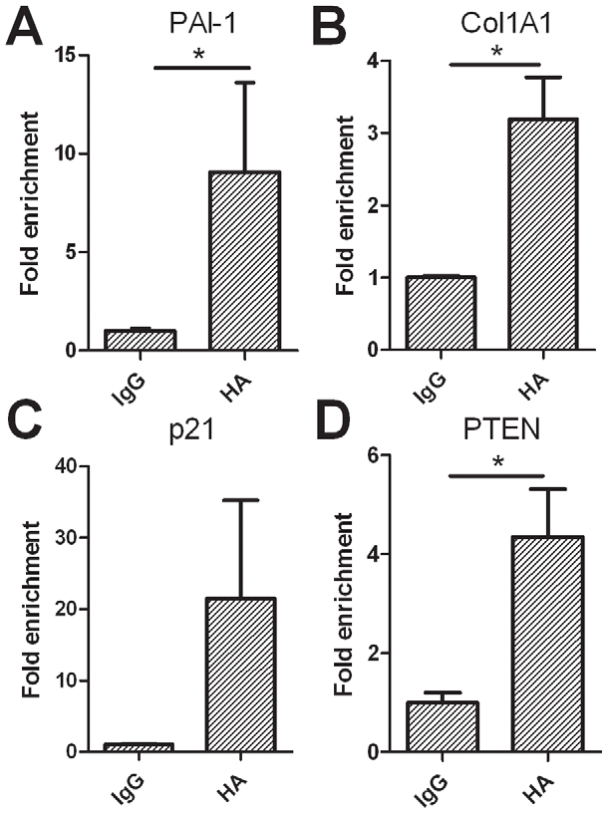
ΔNp73 interacts directly with SBEs in vivo. Chromatin Immunoprecipitation (ChIP) of ΔNp73 binding with SBE. The relative amount of ΔNp73 associated DNA as pulled down with an antibody directed against HA, is represented as a fold enrichment compared to pull-down with IgG (background). Gene enrichment was quantified by qPCR using primers specific for the promoter regions of A) PAI-1, B) Col1a1 and C) p21^WAF^ within the SBEs. Primers specific for PTEN (D) were used as a positive control. Pulldown antibody is shown on the x-axis, with y-axis showing fold enrichment ± SEM. * represents p-value>0.05.

While the DNAP assay demonstrated a direct interaction between ΔNp73 and TGF-β target promoters in vitro, we sought to determine whether this interaction also occurs in vivo. To this end, chromatin immunoprecipitation (ChIP) was performed in TGF- β stimulated Hep3B cells transiently overexpressing recombinant HA tagged ΔNp73. An antibody against HA was used, as the PAN-p73 antibody interaction was insufficient to demonstrate direct binding. Following pulldown with the HA antibody, the interacting DNA was quantitated with qPCR using primers specific for the promoter region of PTEN, PAI-1, p21 and Col1a1 and compared to IgG as background. PTEN serves as a positive control as a previous study [51] has demonstrated that ΔNp73 binds directly to its promoter region. Indeed PTEN showed a 4.35±0.97 (p = 0.0048; Figure 5D) fold enrichment compared to IgG. Further we observed, a significant *in vivo* interaction of ΔNp73 with the SBE in PAI-1 (9.05±4.56; p = 0.038; Figure 5A) and Col1a1 (3.19±0.58; p = 0.003; Figure 5B). P21 showed the highest enrichment, however this was just not significant (21.52±13.74; p = 0.06, Figure 5C). Binding of ΔNp73 was not observed in unstimulated Hep3B cells suggesting that the interaction is indeed TGF- β mediated (data not shown).

## Discussion

High expression levels of ΔNp73 have been shown to strongly correlate with poor survival of cancer patients and ΔNp73 positive tumors show a reduced response to chemotherapy and irradiation (reviewed in [2]).

Whereas we confirmed that ΔNp73 inhibits transactivation activities of p53/p73 on p53 binding element sequences in a dominant-negative fashion as described previously [1]–[3], [6], [31], [38], our results indicate that its action on TGF-β target genes is non-canonical and does not reduce but rather enhance target gene expression. Our data suggest that the latter transactivation activities may require interaction with Smad proteins on Smad Binding Elements (SBEs). ΔNp73 was found to enhance the interaction possibly through binding of Smads to SBEs (and vice versa). This provokes the speculation that induction of TGF-β signaling targets by ΔNp73 could potentially be mediated by the formation of a tertiary ΔNp73-Smad complex at the SBEs that seems to be more efficient in transactivation than a complex of Smads and SBE DNA alone.

Our data combined with previous data by others indicates that further studies are warranted on the role that ΔNp73 might play a role in two major biological processes through which carcinogenesis could be supported. Its inhibition of p53 signaling may prevent apoptosis and cell cycle arrest, supporting growth and survival of cells with increased genetic instability [2], [6], [38]. Its potential concomitant stimulation of TGF-β signaling, which may support invasiveness and metastatic potential of these increasingly more aggressive tumor cells [20]. This may be suggested by the two TGF-β target genes PAI-1 and Col1a1 of which the expression was enhanced by ΔNp73 and which are both directly associated with development and carcinogenesis [23]–[25], [34], [47]–[49]. Together these two mechanistically distinct actions of ΔNp73 would explain the exceptionally strong correlation between high ΔNp73 expression in tumors and poor patient survival [2], [52], [53]. This suggests that ΔNp73 may have additional functions that could play a role not only at high/pathological levels of ΔNp73 in cancer therapy resistance and/or tumor aggressiveness but also in development by stimulating expression of TGF-β signaling targets via a novel, non-canonical pathway.

The assumption that ΔNp73 can enhance the expression of specific TGF-β signaling targets was supported by a number of independent observations in three different cell-lines, using four different luciferase reporters, and a direct interaction determined by ChIP. Whereas all complement each other and together generate a comprehensive picture (explained in detail in Text S1), we are aware that some of the effects although highly significant are small. In all, we do not show proof that any of this may translate to biologically relevant effects in vivo. Therefore, the *in vivo* importance of this cooperation remains to be explored. However, since the combined data are highly congruent it is not unlikely that if these small effect chronically persist in vivo, they may exert biological effects. Although this is beyond the scope of this report, crossing recently published ΔNp73 specific mice [9], [11] with mice deficient in core TGF-β signaling components may help to further establish the exact biological importance of ΔNp73 in TGF-β signaling.

## Supporting Information

Figure S1 **Expression of p53 and p73 in Hep3B, Hek293 and MDA-MB-468 cells.** Cells were seeded, left untreated or treated with 1 ng/ml TGF-β1. 24 hours after treatment, cells were lysed. Lysates were immunoblotted for p53, PAN-p73 and γ-tubulin as loading control. Hek293 cells show high expression of p53 and p73, MDA-MB-468 show moderate expression of p53 and p73 and no expression of p53 and p73 was observed in Hep3B cells. Identification of the ΔNp73α band was done by comparing the band pattern on this blot to HAΔNp73α transfected cells (similar to main figure 2D). No differences in p53 or p73 expression were observed after TGF-β1 treatment.
(PDF)

Figure S2 **Expression of transfected ΔNp73 in Hep3b, Hek293 and MDA-MB-468 cells.** Cells were seeded, transfected with the indicated amount of ΔNp73 and further left untreated or treated with 1 ng/ml TGF-β1. 24 hours after TGF-β1 treatment, cells were lysed. Lysates were immunoblotted with α-HA to detect transfected ΔNp73 and γ-tubulin as loading control. A) Hep3B cells show moderate expression of transfected ΔNp73. B) Hek293 cells show very high expression of transfected ΔNp73, which can even be detected at the very low concentrations used under experimental conditions (10 ng/well). C) MDA-MB-468 cells show very low expression of transfected ΔNp73, it can barely be detected between background bands in 500 ng/well transfected cells in long exposed blots. Note that in figure B, a blot that was clean enough to enhance the signal of α-HA to a level that MDA-MB-468 cells transfected with 500 ng HAΔNp73 do show a clear band, showing that these cells do expess some transfected DNA. (we speculate that Hek293 blots are particularly clean because transfected Hek293 cells express a lot of HAΔNp73, which ensures specific binding and thereby may prevent non-specific bands). No differences in p53 or p73 expression were observed after TGF-β1 treatment.
(PDF)

Figure S3 **Expression of p73 and p53 after transfection of ΔNp73 and TAp73 in Hep3b, Hek293 and MDA-MB-468 cells under experimental conditions.** Cells were seeded at 60% confluence in 24 wells plates, transfected with 10 ng/well ΔNp73 or TAp73 and further left untreated or treated with 1 ng/ml TGF-β1. 24 hours after TGF-β1 treatment, cells were lysed. Lysates were immunoblotted with α-PANp73 to detect all p73, α-HA to detect transfected p73 and γ-tubulin as loading control. Transfected p73 was not detected in Hep3B cells or MDA-MB-468 cells transfected with 10 ng/well (experimental amount). Both transfected ΔNp73 or TAp73 was detected in similar amounts in Hek293 cells. No differences in p53 or p73 expression were observed after TGF-β1 treatment.
(PDF)

Figure S4 **Expression of Smad4 in Hep3b, Hek293 and MDA-MB-468 cells under experimental conditions.** Cells were seeded at 60% confluence in 24 wells plates, transfected with 400 ng/well Luciferase reporter plasmid, 100 ng/well empty vector or Smad4 expressing vector and further left untreated or treated with 1 ng/ml TGF-β1. 24 hours after TGF-β1 treatment, cells were lysed. Lysates were immunoblotted with α-Smad4 to detect all Smad4 (endogenous and transfected) and γ-tubulin as loading control. Transfected Smad4 was clearly detected in Hek293 cells, after long exposure Hek293 also show an endogenous Smad4 band in a high exposed blot. Smad4 was barely detected in transfected Hep3b cells (not visible in these exposures) and no Smad4 was observed in MDA-MB-468 showing that Smad4 expression in transfected MDA-MB-468 cells is lower than endogenous Smad4 in Hek293 cells. No differences were observed after TGF-β1 treatment.
(PDF)

Figure S5 **Tetracycline inducible HAΔNp73α Hek293 cells.** Tetracycline inducible HAΔNp73α Hek293 cells cells were generated. Cells were seeded at 60% confluence in 6- wells plates, half was left untreated and half was treated with 1 µg/ml tetracycline for 24 hours after which cells were lysed. Lysates were immunoblotted with α-HA to detect induction of ΔNp73 and with α-GAPDH as loading control.
(PDF)

Figure S6 **No interactions between ΔNp73 and endogenous Smad3 proteins in extracts of soluble proteins in the absence of DNA.** Immunoprecipitation of Endogenous Smad3 with α-HA antibody in Hek293ΔNp73 cells, left untreated, treated with 1 ng/ml TGF-β1, ΔNp73 induced (+1 µg/ml tetracycline) or both. Extracts of input and of Ip were reacted with α-HA or α-Smad3 antibody. The band in IP samples with Smad3 antibody are non-specific bands which run lower than the Smad3 specific band (visible only in the input samples). Endogenous Smad3 was not detected in IP samples.
(PDF)

Text S1 **Used cell lines and reporter constructs.**
(PDF)
