# Supplementary figures and images for: ΔNp73 Enhances Promoter Activity of TGF-β Induced Genes
Source: PLoS One. 2012 Dec 7;7(12):e50815. doi: 10.1371/journal.pone.0050815 (PMC3517593; doi:10.1371/journal.pone.0050815)

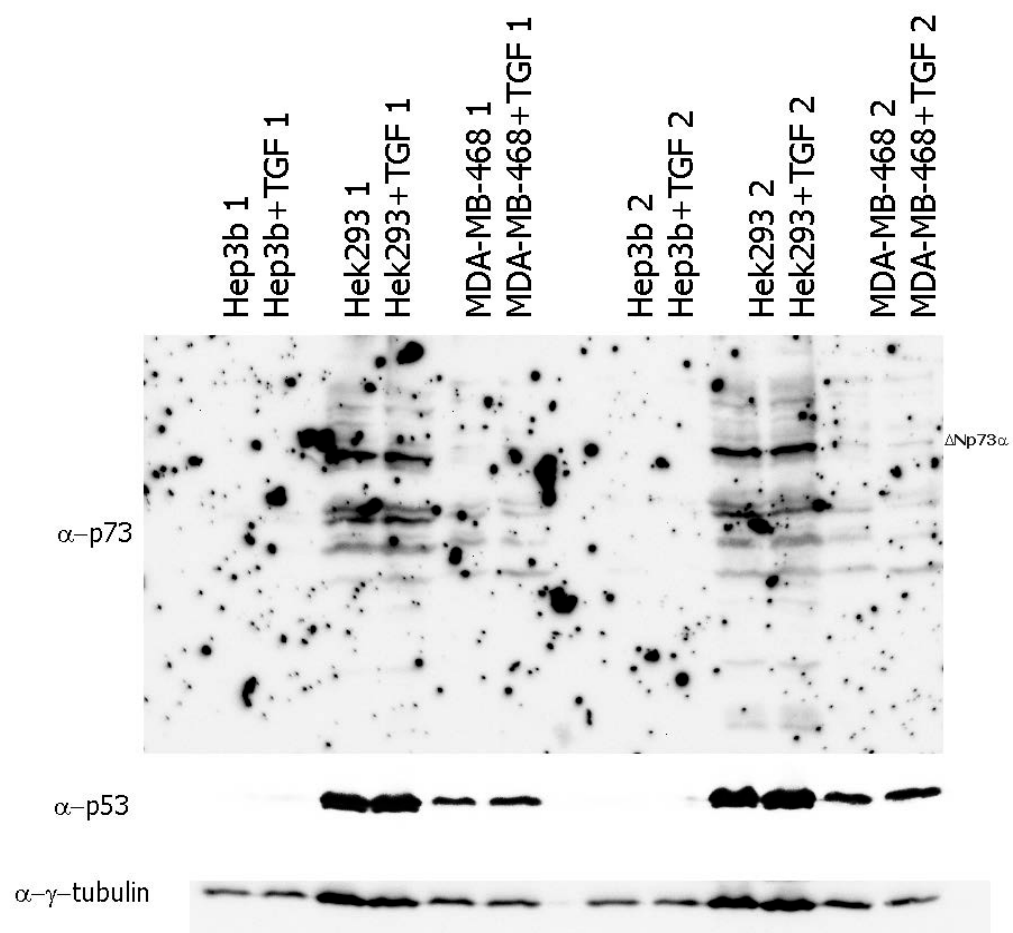

Supplement: Figure S1 — Expression of p53 and p73 in Hep3B, Hek293 and MDA-MB-468 cells. Cells were seeded, left untreated or treated with 1 ng/ml TGF-β1. 24 hours after treatment, cells were lysed. Lysates were immunoblotted for p53, PAN-p73 and γ-tubulin as loading control. Hek293 cells show high expression of p53 and p73, MDA-MB-468 show moderate expression of p53 and p73 and no expression of p53 and p73 was observed in Hep3B cells. Identification of the ΔNp73α band was done by comparing the band pattern on this blot to HAΔNp73α transfected cells (similar to main figure 2D). No differences in p53 or p73 expression were observed after TGF-β1 treatment. (PDF) [file pone.0050815.s001.pdf]

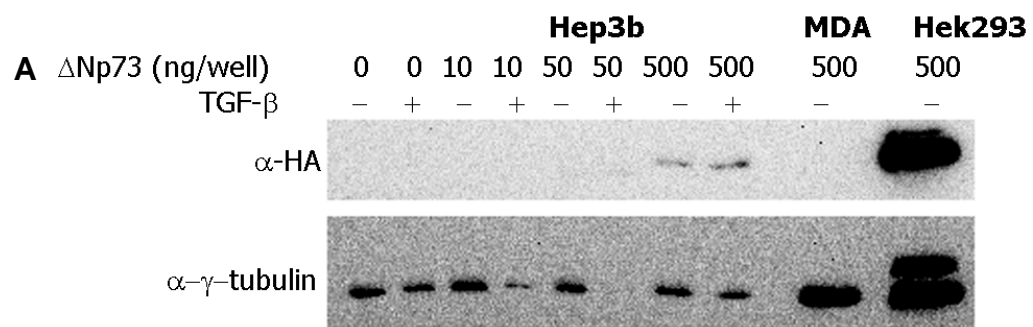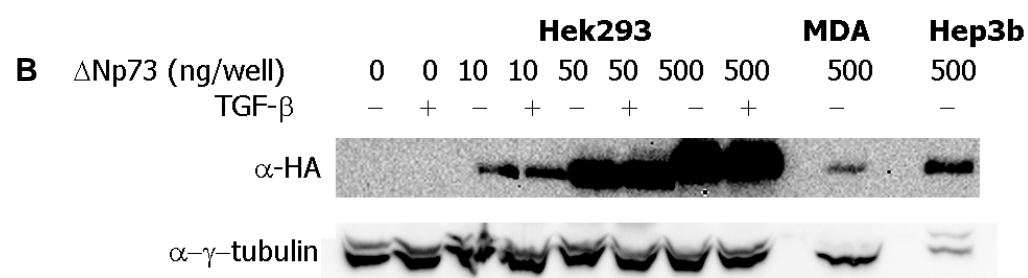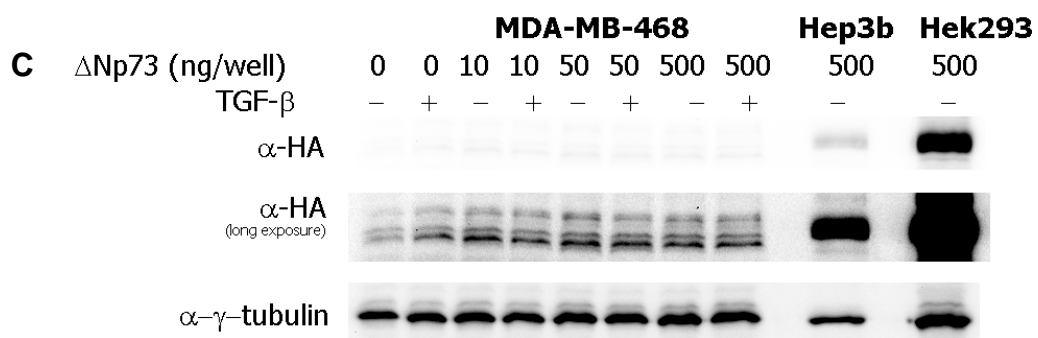

Supplement: Figure S2 — Expression of transfected ΔNp73 in Hep3b, Hek293 and MDA-MB-468 cells. Cells were seeded, transfected with the indicated amount of ΔNp73 and further left untreated or treated with 1 ng/ml TGF-β1. 24 hours after TGF-β1 treatment, cells were lysed. Lysates were immunoblotted with α-HA to detect transfected ΔNp73 and γ-tubulin as loading control. A) Hep3B cells show moderate expression of transfected ΔNp73. B) Hek293 cells show very high expression of transfected ΔNp73, which can even be detected at the very low concentrations used under experimental conditions (10 ng/well). C) MDA-MB-468 cells show very low expression of transfected ΔNp73, it can barely be detected between background bands in 500 ng/well transfected cells in long exposed blots. Note that in figure B, a blot that was clean enough to enhance the signal of α-HA to a level that MDA-MB-468 cells transfected with 500 ng HAΔNp73 do show a clear band, showing that these cells do expess some transfected DNA. (we speculate that Hek293 blots are particularly clean because transfected Hek293 cells express a lot of HAΔNp73, which ensures specific binding and thereby may prevent non-specific bands). No differences in p53 or p73 expression were observed after TGF-β1 treatment. (PDF) [file pone.0050815.s002.pdf]

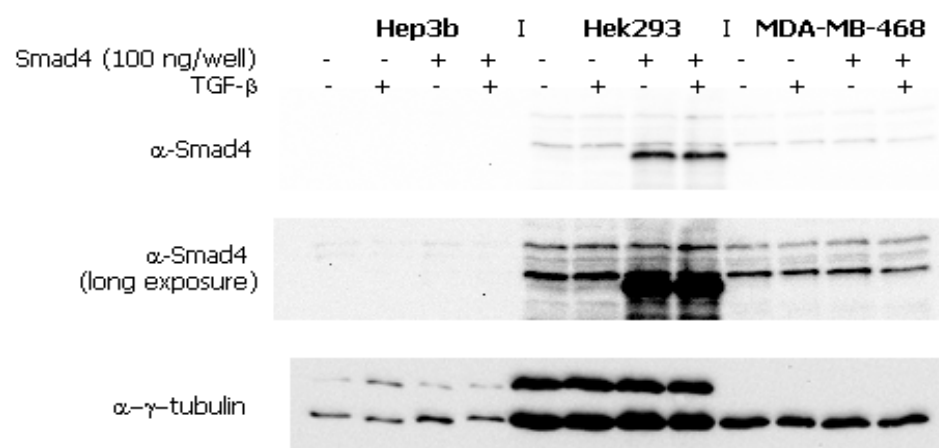

Supplement: Figure S4 — Expression of Smad4 in Hep3b, Hek293 and MDA-MB-468 cells under experimental conditions. Cells were seeded at 60% confluence in 24 wells plates, transfected with 400 ng/well Luciferase reporter plasmid, 100 ng/well empty vector or Smad4 expressing vector and further left untreated or treated with 1 ng/ml TGF-β1. 24 hours after TGF-β1 treatment, cells were lysed. Lysates were immunoblotted with α-Smad4 to detect all Smad4 (endogenous and transfected) and γ-tubulin as loading control. Transfected Smad4 was clearly detected in Hek293 cells, after long exposure Hek293 also show an endogenous Smad4 band in a high exposed blot. Smad4 was barely detected in transfected Hep3b cells (not visible in these exposures) and no Smad4 was observed in MDA-MB-468 showing that Smad4 expression in transfected MDA-MB-468 cells is lower than endogenous Smad4 in Hek293 cells. No differences were observed after TGF-β1 treatment. (PDF) [file pone.0050815.s004.pdf]

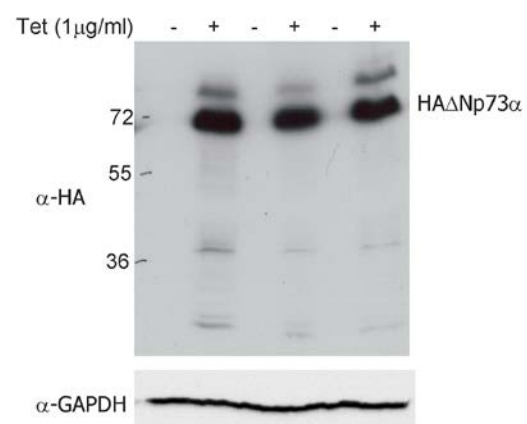

Supplement: Figure S5 — Tetracycline inducible HAΔNp73α Hek293 cells. Tetracycline inducible HAΔNp73α Hek293 cells cells were generated. Cells were seeded at 60% confluence in 6- wells plates, half was left untreated and half was treated with 1 µg/ml tetracycline for 24 hours after which cells were lysed. Lysates were immunoblotted with α-HA to detect induction of ΔNp73 and with α-GAPDH as loading control. (PDF) [file pone.0050815.s005.pdf]

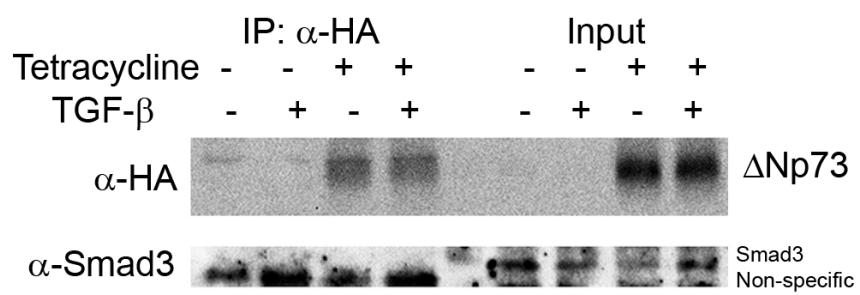

Supplement: Figure S6 — No interactions between ΔNp73 and endogenous Smad3 proteins in extracts of soluble proteins in the absence of DNA. Immunoprecipitation of Endogenous Smad3 with α-HA antibody in Hek293ΔNp73 cells, left untreated, treated with 1 ng/ml TGF-β1, ΔNp73 induced (+1 µg/ml tetracycline) or both. Extracts of input and of Ip were reacted with α-HA or α-Smad3 antibody. The band in IP samples with Smad3 antibody are non-specific bands which run lower than the Smad3 specific band (visible only in the input samples). Endogenous Smad3 was not detected in IP samples. (PDF) [file pone.0050815.s006.pdf]
