# Supplementary material for: ΔNp73 Enhances Promoter Activity of TGF-β Induced Genes
Source: PLoS One. 2012 Dec 7;7(12):e50815. doi: 10.1371/journal.pone.0050815 (PMC3517593; doi:10.1371/journal.pone.0050815)
Supplement: Figure S3 — Expression of p73 and p53 after transfection of ΔNp73 and TAp73 in Hep3b, Hek293 and MDA-MB-468 cells under experimental conditions. Cells were seeded at 60% confluence in 24 wells plates, transfected with 10 ng/well ΔNp73 or TAp73 and further left untreated or treated with 1 ng/ml TGF-β1. 24 hours after TGF-β1 treatment, cells were lysed. Lysates were immunoblotted with α-PANp73 to detect all p73, α-HA to detect transfected p73 and γ-tubulin as loading control. Transfected p73 was not detected in Hep3B cells or MDA-MB-468 cells transfected with 10 ng/well (experimental amount). Both transfected ΔNp73 or TAp73 was detected in similar amounts in Hek293 cells. No differences in p53 or p73 expression were observed after TGF-β1 treatment. (PDF) [file pone.0050815.s003.pdf]

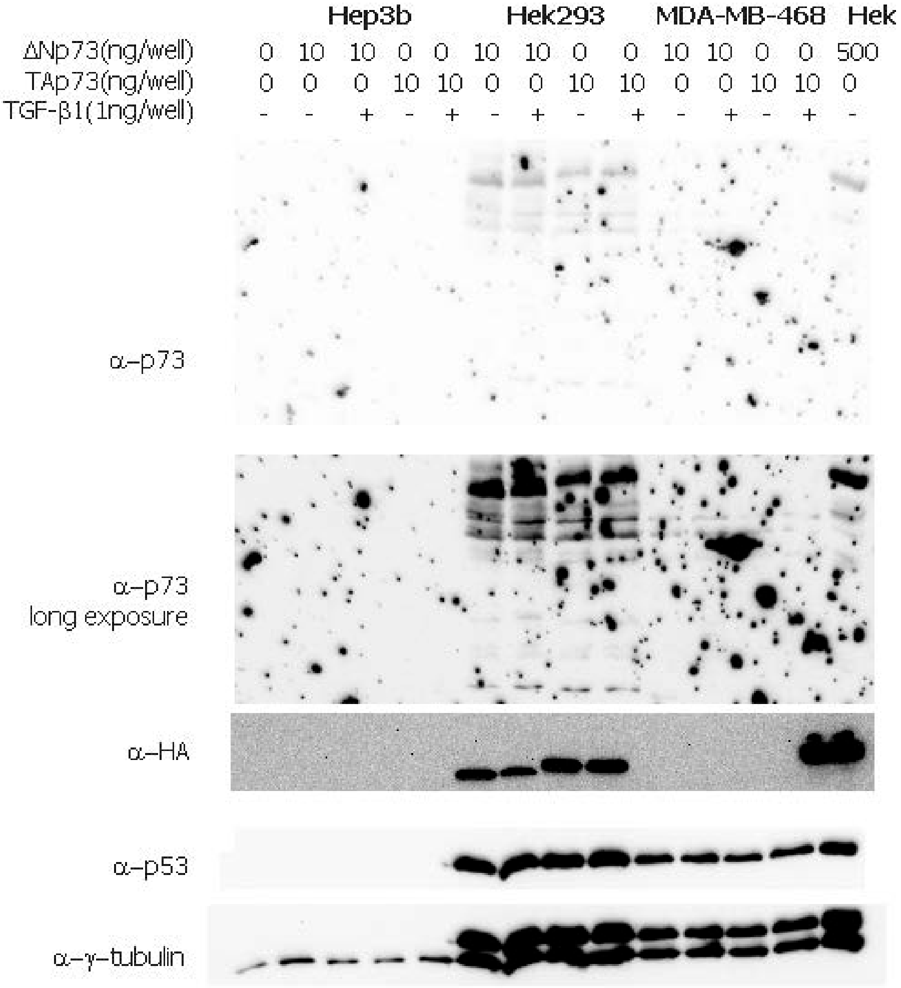

Hep3b
Hek293
MDA-MB-468
Hek
ΔNp73(ng/well) 0 10 10 0 0 10 10 0 0 10 10 0 0 500
TAp73(ng/well) 0 0 0 10 10 0 0 10 10 0 0 10 10 0
TGF-β1(1ng/well) - - + - + - + - + - + - + -
α–p73
α–p73
long exposure
α–HA
α–p53
α–γ–tubulin
