# Supplementary material for: ΔNp73 Enhances Promoter Activity of TGF-β Induced Genes
Source: PLoS One. 2012 Dec 7;7(12):e50815. doi: 10.1371/journal.pone.0050815 (PMC3517593; doi:10.1371/journal.pone.0050815)
Supplement: Text S1 — Used cell lines and reporter constructs. (PDF) [file pone.0050815.s007.pdf]

Supporting text S1

Used cell lines and reporter constructs

To study the mechanism behind a possible role of ΔNp73 in TGF-β signaling we used three different cell-lines and four different luciferase reporters, all for different purposes to generate a comprehensive picture. An overview of specific reasons for using these cell-lines and reporters is given here in detail.

First, the Hep3B cells were used because they do not express p53 and p73 (figure S1 and (1, 2)) and express modest levels of transfected DNA (figure S2a), which allowed us to study TGF-β responses to ΔNp73 without interference of endogenous p53 or p73 under transfection conditions that did not result in over-the-top expression of transfected DNA.

Second, the Hek293 cells were used because they do express endogenous p53 and p73 including ΔNp73 (figures S1 and 2D) and express very high levels of transfected DNA (figure S2b), which allowed us to; 1) study if pathological high expression of endogenous p53 and p73 significantly alters the effect of ΔNp73 on TGF-β signaling compared to Hep3B cells 2) to study if downregulation of ΔNp73 reduces TGF-β signaling and 3) to generate the high levels of ectopic DNA required for biochemical assays. In addition, the Hek293 cells were used to generate a tetracycline-inducible ΔNp73 expression cell line to study the impact of ΔNp73 expression on the induction of endogenous TGF-β signaling targets (figure S5).

Third, the MDA-MB-468 cells were used to study the requirement for an intact Smad signaling machinery for the ΔNp73 induced enhancement of TGF-β signaling because these cells are deficient for Smad4 (3, 4). A drawback of this cell line was that transfection efficiency was very low (figure S2).

In addition to different cell lines we also used different luciferase reporter constructs to elucidate the potential role of ΔNp73 in TGF-β signaling.

First, we used the PAI-luc reporter, the PAI-1 promoter contains all binding sites for transcription factors normally present in the PAI-1 promoter including both a p53 binding element and three Smad binding elements. This construct is normally used to study the cooperation between p53 family members and TGF-β signaling, however it can also be used to study TGF-β responses alone quite efficiently due to the three SBEs and its high read-out (1, 5, 6).

Second, the p21-luc construct, the p21 promoter contains all binding sites for transcription factors normally present in the p21 promoter including two p53 binding elements and one Smad binding element (7). This construct was used to study the dominant-negative character of our transfected ΔNp73.

Third, we used the PAI1-luc with mutated p53 binding-site, this promoter has all the binding-sites present in the PAI-luc construct but the p53 binding-site is mutated and thus not functional (1). This construct was used to study which of the p73 forms rely on the p53 binding element for increase of PAI1 expression.

Fourth, the SBE-luc construct, this promoter is comprised only of Smad Binding Elements. This synthetic construct was used in most reporter experiments because it responds to TGF-β signals without any requirement for or interference of other signals and thus gives the “most pure” TGF-β response (8). A drawback of this construct is that it has a low readout and can thus be used only in cells with a reasonable uptake of plasmid DNA during transfection (unfortunately not in MDA-MB-468 cells).

references

1. Hageman, J., Eggen, B. J., Rozema, T., Damman, K., Kampinga, H. H., and Coppes, R. P. Radiation and transforming growth factor-beta cooperate in transcriptional activation of the profibrotic plasminogen activator inhibitor-1 gene. Clin Cancer Res, *11:* 5956-5964, 2005.

2. Lasfer, M., Davenne, L., Vadrot, N., Alexia, C., Sadji-Ouatas, Z., Bringuier, A. F., Feldmann, G., Pessayre, D., and Reyl-Desmars, F. Protein kinase PKC delta and c-Abl are required for mitochondrial apoptosis induction by genotoxic stress in the absence of p53, p73 and Fas receptor. FEBS Lett, *580:* 2547-2552, 2006.
3. de Caestecker, M. P., Hemmati, P., Larisch-Bloch, S., Ajmera, R., Roberts, A. B., and Lechleider, R. J. Characterization of functional domains within Smad4/DPC4. J Biol Chem, *272:* 13690-13696, 1997.
4. de Winter, J. P., Roelen, B. A., ten Dijke, P., van der Burg, B., and van den Eijnden-van Raaij, A. J. DPC4 (SMAD4) mediates transforming growth factor-beta1 (TGF-beta1) induced growth inhibition and transcriptional response in breast tumour cells. Oncogene, *14:* 1891-1899, 1997.
5. Cordenonsi, M., Dupont, S., Maretto, S., Insinga, A., Imbriano, C., and Piccolo, S. Links between tumor suppressors: p53 is required for TGF-beta gene responses by cooperating with Smads. Cell, *113:* 301-314, 2003.
6. Niemantsverdriet, M., de Jong, E., Langendijk, J. A., Kampinga, H. H., and Coppes, R. P. Synergistic induction of profibrotic PAI-1 by TGF-beta and radiation depends on p53. Radiother Oncol, *97:* 33-35, 2010.
7. Niemantsverdriet, M., Jongmans, W., and Backendorf, C. Radiation response and cell cycle regulation of p53 rescued malignant keratinocytes. Exp Cell Res, *310:* 237-247, 2005.
8. Jonk, L. J., Itoh, S., Heldin, C. H., ten Dijke, P., and Kruijer, W. Identification and functional characterization of a Smad binding element (SBE) in the JunB promoter that acts as a transforming growth factor-beta, activin, and bone morphogenetic protein-inducible enhancer. J Biol Chem, *273:* 21145-21152, 1998.
